# The Diagnostic Dilemma of “The Great Imitator”: Heart and Cerebral Involvement of Lupus Manifesting as Bilateral Upper and Lower Extremity Weakness

**DOI:** 10.1155/2023/6676357

**Published:** 2023-10-10

**Authors:** Alexander Santos, Catrina Kure, Cesar Sanchez, Phillip Gross

**Affiliations:** Northeast Georgia Medical Center, Gainesville, USA

## Abstract

**Background:**

Systemic lupus erythematous (SLE) is an autoimmune condition which can cause complex, multiorgan dysfunction. This autoimmune disease is caused by the production of antinuclear antibodies which allows this disease to target virtually any organ in the human body. When a patient experiences an unpredictable worsening of disease activity, it is generally considered a lupus flare. Organ dysfunction due to a lupus flare tends to manifest as separate events in the literature and rarely do we witness multiple compounding organ failures during a lupus flare. If we do witness organ dysfunction and failure, rarely do we see cardiac and cerebral involvement. Typically, patients take immunosuppressants for a long term to avoid the patient's disease process from worsening and to provide prophylaxis from a flare to occur. Despite the availability in preventive strategies, some patients will have increased disease activity multiple times throughout their lifetime and will need increases in their medication doses or changes to their regimen. Some flares can be managed in the clinic, but more severe ones may be life-threatening that they require intravenous medications and hospitalization to achieve remission. In the following case, we see a patient with a past medical history of SLE on multiple immunosuppressants who arrived at the hospital with acute, bilateral weakness of the upper and lower extremities. It was later determined via various imaging and laboratory testing that she was having an SLE flare that was directly causing myocarditis which progressed to global ischemia of the brain via myocardial hypoperfusion. She experienced substantial recovery from her flare with treatment with high-dose, intravenous corticosteroids. *Case Report*. A 27-year-old female with a 2-year history of lupus and a 1-week history of paroxysmal atrial fibrillation presented with three days of bilateral focal neurological deficits in the arms and legs. She was found to have ischemic cardiac and neurologic manifestations during her hospital stay.

**Conclusion:**

Our patient presented with reversible focal neurological deficits, elevated high-sensitive troponin levels, and high lupus serum antibodies who showed significant improvement after the introduction of high-dose steroids. This case recommends keeping a large differential and to not discount patients' past comorbidities for causing atypical symptomatology.

## 1. Introduction/Background

Systemic lupus erythematous is a connective tissue disorder which can affect any organ with varying severity. Sometimes known as “the great imitator,” SLE manifests in a myriad of ways, having the potential to cause deadly neurological and cardiovascular complications [1–4]. According to the American College of Rheumatology, neuropsychiatric systemic lupus erythematous (NPSLE) is divided into 19 manifestations, most commonly headache, cognitive dysfunction, cerebrovascular disease (CVD), psychosis, and delirium [5, 6]. Most patients with lupus who have had CVD had subsequent irreversible focal neurological deficits, partly because these pathologies are caused by arterial occlusion or hemorrhage. A few case reports highlighted the rarity of reversible neurological deficits in SLE [7].

Cardiac manifestations are seen in 50% of patients with SLE over a lifetime. The most common manifestation is pericarditis, which is seen in about 25% of those with SLE over a lifetime. A lesser common one, myocarditis, is seen in as low as 9% of those with SLE and cardiac problems [2, 8, 9]. Simultaneous focal neurological deficits and myocarditis have not been reported in the literature to our knowledge.

Our aim in this report is to describe the atypical presentation of neurological and cardiac dysfunction in a patient with active SLE, as well as mentioned how this case differs from those seen in the literature so far. We also highlight some of the available instruments for measuring SLE activity.

We herein present a case report of a patient with a 2-year history of SLE and a 1-week history of paroxysmal atrial fibrillation who demonstrates new focal neurological deficits bilaterally and myocarditis.

## 2. Case Presentation

**Figure 1:**
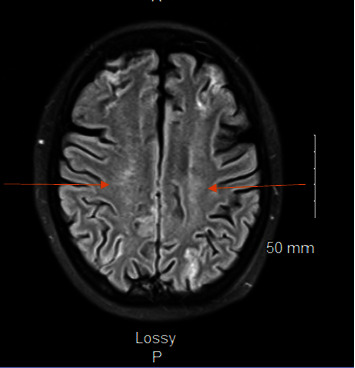
MRI of patient's brain showing global ischemia. This brain image shows an image cut from the patient's MRI that was ordered as with and without contrast of the brain. Global ischemic changes are noted by the orange arrows noted at the watershed areas.

**Figure 2:**
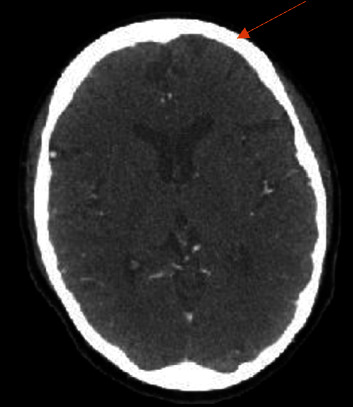
Computed tomography angiography head and neck of the patient showing ischemia of the brain. This image is of a computed tomography angiography head and neck. The orange arrow notes mild hypoattenuation in the high left frontal convexity.

A 27-year-old female with a 2-year history of lupus and a 1-week history of paroxysmal atrial fibrillation presented with three days of bilateral focal neurological deficits in the arms and legs. She denies any history of thrombosis in the past. Her home medications included mycophenolate 250 mg twice daily, hydroxychloroquine 200 mg twice daily, metoprolol 50 mg twice daily, and apixaban 5 mg twice daily. Upon presentation, she was hemodynamically stable and afebrile. Using the medical research council scale for muscle strength (MRC) on her physical exam, she had 1/5 strength in her right upper and lower extremities and 2/5 strength in her left upper and lower extremities. Upon further examination, her cranial nerves II through XII were intact and light touch was intact in the upper and lower extremities. Speech was normal, and she was alert orientated to herself, location, and time. Her weakness of the right upper and lower extremity was profound and affected her abductors, flexors, extensors, and those that provide internal and external rotation. As for her left upper extremity, her weakness was in the same nature but at a lesser degree. The high-sensitive troponin level was 2781 ng/ml, and the ECG showed normal sinus rhythm with nonspecific ST wave abnormalities stable from previous ECG. The patient was started on clopidogrel 75 mg and atorvastatin 40 mg daily and continued apixaban and metoprolol. A computed tomography angiography (CTA) of the brain showed hypoattenuation in the left frontal convexity. A magnetic resonance imaging (MRI) brain with contrast was notable for acute ischemic changes in the watershed area among the anterior, middle, and posterior cerebral arteries (Figure 1). CTA of the patient's brain showed ischemia via subtle hypoattenuation on imaging of the high left frontal convexity in the brain (Figure 2), accessed 2022, image taken Nov 2021, and site: Gsv Ed Ct Imaging. An MRI with and without contrast of the cervical spine was completed which noted neither areas of enhancement nor any areas of stenosis at the cervical joints and spinal cord. As her cervical spine was found to be unremarkable on MRI, the rest of spine was not completed, image taken November 2021, and site: Gsv Mc Mr Imaging. A transthoracic echocardiogram (TTE) with a bubble study was negative for coronary thrombosis. The cardiology team decided on not moving forward with a transesophageal echocardiogram (TEE) as her symptomatology was unlikely from thrombosis. By day two, the patient was still not improving and her high-sensitive troponin level continued to trend upwards. Cardiology was consulted for consideration of ischemic evaluation given the peaked high-sensitive troponin level of 5472 ng/ml. A CT PET of her heart was ordered which did not show a perfusion defect, ruling out a myocardial infarction. Neurology was consulted and recommended holding apixaban and starting heparin in case of stroke etiology, as well as to order a lumbar puncture. The patient had a lumbar puncture while in the hospital. Her cerebrospinal fluid (CSF) noted protein of 30.6 mg/dL, CSF glucose of 51 mg/dL, 82 RBC cells/mm3, 1 lymphocyte cell/mm3, and 10 monocytes cells/mm3, and its appearance was clear. With this information, all serological, cytological, and biochemical parameters were normal, ruling out an infectious process, tumors, or neurological illness. Her creatine kinase was found to range from 29 U/L to 46 U/L, and thus her symptoms were unlikely a myositis. The patient continued to show no improvement.

Rheumatology was consulted, and they stated that the overall clinical picture could be a lupus flare. Rheumatology recommended 1.0 g solumedrol for three days followed by prednisone 60 mg daily as well as a lupus flare work-up. ANA was positive. Sjogren's antibody, DNA DS antibody, and atypical p-ANCA antibody were elevated. SS-A was 3.7 U (<0.2 is normal), SS-B < 0.2, Sm Ab IgG < 0.2 U, RNP Ab IgG < 0.2 U, Scl 70 Ab IgG *S* < 0.2 U, Jo 1 Ab IgG *S* < 0.2 U, C3 was 110.30, and C4 was 26.80 compared to 82.70 and 19.90, respectively, two months earlier. The patients' complement levels were taken after the start of her intensive immunosuppression therapy and thus could cause her results to be falsely normal. DS-DNA Ab was above upper limit of test, >1000, without a previous value for comparison. Patients' lupus anticoagulant was unremarkable. Cardiolipin antibody IgA < 10 APL (normal: 0 to 11), cardiolipin antibody IgG was 13 GPL (normal: 0 to 14), and cardiolipin antibody IgM was 12 MPL (normal: 0 to 12). Protein electrophoresis showed a normal electrophoretic pattern. The patient was continued on mycophenolate with a new regimen of 500 mg BID, hydroxychloroquine 200 mg BID and prednisone 60 mg QD for two weeks followed by a steroid taper of 40 mg for two weeks followed by 20 mg for two weeks and remain on 10 mg daily until her follow-up visit with her rheumatologist.

After reconvening with neurology, they stated that a cause of the stroke-like symptoms was neuropsychiatric systemic lupus erythematosus (NPSLE), a diagnosis of exclusion. After discussion with cardiology and rheumatology, it was thought that the patient's elevated high-sensitive troponin levels were SLE-induced too, most likely being a presentation of lupus myocarditis. The patient showed significant improvement in her signs and symptoms after the steroids were introduced. The strength in both her legs and left arm was 4/5 and then right arm was 3/5.

## 3. Discussion

In this case report, we describe a patient who presented with focal neurologic deficits and myocarditis who demonstrated improvement with the 1.0 g solumedrol for three days. This diagnosis was difficult as her symptoms mimicked a primary cerebrovascular event. We expanded our differentials and considered a rheumatological cause after ruling out a primary cardiac and neurological cause. An increase in the serum titer of anti-dsDNA antibodies and a fall in complement levels such as C3 and C4 are helpful in determining the presence of active lupus [10]. Ribosomal P antibodies in the serum or CSF are highly specific biomarkers for NPSLE, which unfortunately we did not test for [11]. Normal CSF cell counts consist of <5 cells/mm^3^. CSF pleocytosis can be indicative of neuroinflammation from systemic lupus erythematous or also from blood contamination during the procedure. When pleocytosis is reactive, it usually presents with an increase in mononuclear cells. With this information, it is possible that the elevation in the presented patient's monocytes arose from the central nervous system involvement of her disease. We attributed her neurological symptoms to NPSLE after correlating her symptoms with her elevation in anti-dsDNA antibody levels and CSF findings, as well as by observing her response to steroids [12].

In the literature, there are a limited number of reported reversible focal neurological deficits caused by SLE. The pathogenesis of cases of reversible neurological deficits reported have been due to lesions associated with isolated seizures, reversible posterior leukoencephalopathy syndrome (RPLS) which has usually been associated with comorbidities driven by SLE such as hypertension or seizures, cerebral edema secondary to a focal infarct, lesions of acute myelopathy, or thrombotic thrombocytopenic purpura (TTP) [7]. This patient did not present with seizures or have any seizure history, high-intensity lesions indicative of RPLS, evidence of edema, or thrombocytopenia. Acute myelopathy was an unlikely cause of her symptoms, and since her lumbar puncture was insignificant, she still had intact sensation in her extremities, and the deficits could not be attributed to a certain spinal cord segment from the cervical MRI obtained. Those patients in the literature who had seizures, cerebral edema, acute myelopathy, or leukoencephalopathy also had high-intensity lesions on T2 weighted imaging, which was not present in this patient who had global ischemia in watershed regions. All patients with SLE with evidence of reversible neurological deficits attributed to NPSLE had almost complete recovery after steroid therapy was given. The cases even reported that patients with hemiplegia had complete recovery. The neurological findings are distinct from the CVD category defined by AACR as CVD in SLE does not respond to steroids as it is due to occlusion or hemorrhage, which further highlights the rarity of our case [7].

The diagnosis of myocarditis is based on clinical suspicion rather than definitive diagnostic tests, especially in those patients with subclinical heart symptoms. We presumed the diagnosis of myocarditis from her elevated high-sensitive troponins, positive serum labs indicative of a lupus flare, and episode of atrial fibrillation which all showed improvement with the initiation of the 1.0 g of solumedrol. This was also a difficult diagnosis to make, as this patient did not present with the classic signs of chest pain, cardiogenic shock, or shortness of breath usually seen in myocarditis. Based on this and the neurological work-up, the pathogenesis of her NPSLE was made clearer in which the cerebral hypoperfusion was from myocarditis. The pathogenesis of reversible neurological deficits is not outlined in the AACR definition of NPSLE, which highlights the unmet need for an expansion of this definition, especially given the several cases that have occurred and have been reported in the literature so far [2, 8, 13].

Certain disease-measuring instruments such as the Physician Global Assessment (PGA) and the SELENA-SLEDAI are widely used for measuring changes in lupus activity in clinical research, and regular use of these tools in routine clinical care is advocated for in the literature [14, 15]. Unfortunately, the scores for this patient were not calculated in real time. Looking back, this patient had a 12-point increase in her SELENA-SLEDAI score and a 2.5-point increase in her PGA score, classifying her disease activity as severe. This case highlights the significance of considering lupus when atypical symptoms are seen in patients, as well as the available tools for assessing lupus activity. Severe lupus calls for the use of the high-dose steroids which was done in this patient.

The patient showed significant improvement on her second day of steroids and continued to improve the following week. Physical therapy was a significant component to her progression to baseline in addition to the steroids. She was scheduled to go to an acute rehabilitation center to regain her strength and was to follow up with her outpatient rheumatologist for long-term management of SLE.

## 4. Conclusion

Systemic lupus erythematous presents in a variety of ways, from subclinical to overtly detrimental. Our patient presented with reversible focal neurological deficits, elevated high-sensitive troponin levels, and high lupus serum antibodies who showed significant improvement after the introduction of high-dose steroids. This case highlights the importance of considering active lupus as a differential diagnosis for atypical presentations of cardiac and neurological involvement. Reversible focal neurological deficits are a distinct entity from CVD and demyelinating diseases but still fit the criteria for NPSLE. This case also highlights the availability of instruments such as the PGA and SELENA-SLEDAI to help assess worsening lupus activity. As cardiac and neurological manifestations are contributors of overall lupus morbidity and mortality, recognizing atypical manifestations and understanding the available tools are vital in routine patient care.

## Data Availability

Main data came from Northeast Georgia Medical System.
